# Development of a Prediction Model and Risk Score for Self-Assessment and High-Risk Population Identification in Liver Cancer Screening: Prospective Cohort Study

**DOI:** 10.2196/65286

**Published:** 2024-12-30

**Authors:** Xue Li, Youqing Wang, Huizhang Li, Le Wang, Juan Zhu, Chen Yang, Lingbin Du

**Affiliations:** 1 Department of Cancer Prevention, Zhejiang Cancer Hospital, Hangzhou Institute of Medicine, Chinese Academy of Sciences, 1 East Banshan Road, Hangzhou, 310022, China, 86 571-88122219; 2 Department of Ultrasound, Zhejiang Cancer Hospital, Hangzhou Institute of Medicine, Chinese Academy of Sciences, Hangzhou, China

**Keywords:** liver cancer, cancer screening, cancer surveillance, prediction model, early detection, risk score, self-assessment

## Abstract

**Background:**

Liver cancer continues to pose a significant burden in China. To enhance the efficiency of screening, it is crucial to implement population stratification for liver cancer surveillance.

**Objective:**

This study aimed to develop a simple prediction model and risk score for liver cancer screening in the general population, with the goal of improving early detection and survival.

**Methods:**

This population-based cohort study focused on residents aged 40 to 74 years. Participants were enrolled between 2014 and 2019 and were prospectively followed until June 30, 2021. Data were collected through interviews at enrollment. A Cox proportional hazards regression was used to identify predictors and construct the prediction model. A risk score system was developed based on the weighted factors included in the prediction model.

**Results:**

A total of 153,082 study participants (67,586 males and 85,496 females) with a mean age of 55.86 years were included. During 781,125 person-years of follow-up (length of follow-up: median 6.07, IQR 3.07‐7.09 years), 290 individuals were diagnosed with liver cancer. Key factors identified for the prediction model and risk score system included age (hazard ratio [HR] 1.06, 95% CI 1.04‐1.08), sex (male: HR 3.41, 95% CI 2.44‐4.78), education level (medium: HR 0.84, 95% CI 0.61‐1.15; high: HR 0.37, 95% CI 0.17‐0.78), cirrhosis (HR 11.93, 95% CI 7.46‐19.09), diabetes (HR 1.59, 95% CI 1.08‐2.34), and hepatitis B surface antigen (HBsAg) status (positive: HR 3.84, 95% CI 2.38‐6.19; unknown: HR 1.04, 95% CI 0.73‐1.49). The model exhibited excellent discrimination in both the development and validation sets, with areas under the curve (AUC) of 0.802, 0.812, and 0.791 for predicting liver cancer at the 1-, 3-, and 5-year periods in the development set and 0.751, 0.763, and 0.712 in the validation set, respectively. Sensitivity analyses applied to the subgroups of participants without cirrhosis and with a negative or unknown HBsAg status yielded similar performances, with AUCs ranging from 0.707 to 0.831. Calibration plots indicated an excellent agreement between the observed and predicted probabilities of developing liver cancer over the 1-, 3-, and 5-year periods. Compared to the low-risk group, participants in the high-risk and moderate-risk groups had 11.88-fold (95% CI 8.67‐16.27) and 3.51-fold (95% CI 2.58‐4.76) higher risks of liver cancer, respectively. Decision curve analysis demonstrated that the risk score provided a higher net benefit compared to the current strategy. To aid in risk stratification for individual participants, a user-friendly web-based scoring system was developed.

**Conclusions:**

A straightforward liver cancer prediction model was created by incorporating easily accessible variables. This model enables the identification of asymptomatic individuals who should be prioritized for liver cancer screening.

## Introduction

Liver cancer is the sixth most common cancer worldwide and the second leading cause of cancer death in China [1,2]. China accounts for nearly half of the global burden of liver cancer [3], and the 5-year survival rate of liver cancer is 12.1% [4]. The majority of patients with liver cancer are diagnosed at an advanced stage, resulting in a poor prognosis. Therefore, efficient prevention remains a critical public health concern. Early detection of liver cancer improves the likelihood of effective treatment, increases the selection of targeted therapies, prevents disease progression, and ultimately improves prognosis. Screening plays a vital role in reducing the burden of liver cancer, and further exploration of optimal population screening strategies is warranted.

Currently, most guidelines consider cirrhosis and infection with the *Hepatitis B virus* (HBV) or the *Hepatitis C virus* (HCV) as risk factors for liver cancer, and screening is recommended for individuals with these conditions. However, due to the effective control of HBV infection, the availability of antiviral treatments, the reduced consumption of aflatoxin-contaminated food, the rising prevalence of metabolic diseases, and an aging population, the pattern of risk factors has changed over the past few decades in China. Therefore, liver cancer risk surveillance and screening strategies for the general population require further evidence and preventive measures. Previous liver cancer prediction models have been limited by focusing on specific populations (such as those with cirrhosis [5,6], HBV infection [7-11], HCV infection [6,12,13] or chronic hepatitis with different etiologies [14]) or relying on clinical or laboratory indicators [15-18]. A simplified and personalized liver cancer risk assessment that incorporates accessible factors will benefit the general population by providing early warnings and enabling timely screening. In this study, we used data from a large-scale, longitudinal follow-up study to develop a prediction model and a user-friendly risk scoring system to assist in liver cancer surveillance.

## Methods

### Data Source and Participants

Data were obtained from a multicenter, population-based cohort study conducted within the framework of the Cancer Screening Program in Urban China (CanSPUC) [19]. Briefly, the CanSPUC is an ongoing, nonprofit, nationwide cancer screening program targeting the 5 most prevalent cancers: liver cancer, lung cancer, breast cancer, upper gastrointestinal cancer, and colorectal cancer. Residents aged 40 to 74 years living in selected communities of the participating cities were recruited through phone calls and personal contact. After signing written informed consent, all eligible participants were interviewed by trained staff to collect data on demographic characteristics, lifestyle factors, general health, and family history of cancer. Participants also underwent a simple physical assessment, including measurements of height and weight, as well as clinical examinations for one or more cancer screenings if necessary, according to the CanSPUC protocol [19].

We used data from the CanSPUC collected between January 2014, and July 2019, in Zhejiang Province, which included 4 cities: Hangzhou, Ningbo, Quzhou, and Jinhua. Participants with a history of cancer diagnosis were excluded from the study, resulting in 153,082 eligible participants for analysis. The time to liver cancer occurrence was calculated from the cohort entry date until the occurrence of liver cancer, death, or administrative censoring (June 30, 2021), whichever came first.

### Ethical Considerations

This study was approved by the Ethics Committees of the China National Cancer Center/Cancer Hospital, the Chinese Academy of Medical Sciences, and Peking Union Medical College (approval number 15-070/997), as well as the Ethics Committee of Zhejiang Cancer Hospital (approval number IRB-2022‐271). The original consent covered secondary analysis without additional consent or compensation, and all data was anonymized.

### Outcome, Variables, and Quality Control

The primary outcome was liver cancer incidence. All new cases of liver cancer in the study were identified through the cancer registry system, with a histologically confirmed diagnosis according to the *International Statistical Classification of Diseases, Tenth Revision* and were coded as C22. The outcome data were retrieved from a national cancer registry system and have been extensively used to assess the disease burden both regionally and nationally in China, as well as globally [20] and for other research purposes [19,21,22]. Covariates from the baseline survey included demographic characteristics (age, sex, height, weight, and education level), lifestyle factors (smoking status, alcohol consumption, frequency of exercise, and occupational exposure to hazardous substances), items assessing general health (chronic respiratory diseases, digestive diseases, hepatobiliary diseases, hypertension, diabetes, and hyperlipidemia), and a family history of cancer. Education was classified as low (primary school or below), medium (junior or senior high school), and high (undergraduate or higher). BMI was calculated based on height and weight. Smokers were defined as individuals who had previously smoked or were currently smoking tobacco more than once per day for at least 6 months. Alcohol consumption was defined as drinking at least once a week for more than 6 months. Frequent exercise was defined as engaging in physical activity at least 3 times per week, with each session lasting more than 30 minutes. Occupational exposure to hazardous substances included exposures to asbestos, rubber, dust, pesticide, radiation, beryllium, uranium, or radon for at least 1 year. Hepatobiliary diseases included chronic HBV or HCV infection, cirrhosis, history of schistosome infection, fatty liver disease, and gallstones. Paper-based or computer-based standardized questionnaires were used to collect information, and trained study staff reviewed and entered the collected data into the data management system. A thorough consistency check was conducted, and any identified inconsistencies were rectified by referring back to the original records. All data were transmitted to the Central Data Management Team at the National Cancer Center of China, where the databases were constructed and analyzed.

### Statistical Analysis

The baseline characteristics of the study population were described as frequency and percentage for categorical variables and as mean and SD or median and IQR for continuous variables. A univariable Cox regression model was used to explore potential factors associated with liver cancer. A multivariable Cox regression model with backward selection was used to identify the variables that were incorporated in the prediction model. Hazard ratios (HR) and 95% CIs were calculated. Time-dependent receiver operating characteristic (ROC) curves and the area under the curve (AUC) were used to assess the performance of the models. Calibration plots were used to assess the agreement between the predicted probability of remaining liver cancer-free (as calculated by the model) and the observed outcomes. The risk score was created by summing the weighted factors incorporated in the prediction model. The score of age was calculated by subtracting 40 from the individual’s age and then multiplying by the β coefficient from the multivariable Cox regression model. The score for categorical variables was defined as the weighted β coefficient from the multivariable Cox regression model, with the reference group assigned a score of 0. The final score for each individual was generated by summing the weighted factors together as follows: risk score = factor 1 score + factor 2 score + factor 3 score + … + factor n score. The risk score was subsequently standardized to a scale of 1‐100 using the following formula: (score – minimum)/(maximum – minimum). X-tile plots were used to generate two optimal cutoff values to separate participants into low-, moderate-, and high-risk groups [23]. Additionally, decision curve analysis was used to compare the net benefit of our model with that of the CanSPUC strategy. All statistical analyses were performed using the statistical software R version 4.2.1 (IBM Corp). All tests were 2-sided, and a *P* value of <.05 was considered statistically significant.

## Results

### Basic Characteristics and Factors Associated With Liver Cancer

Among the 153,082 study participants (67,586 males and 85,496 females) with a mean age of 55.86 years, 290 individuals were diagnosed with liver cancer over 781,125 person-years of follow-up (length of follow up: median 6.07, IQR 3.07‐7.09 years). The baseline characteristics are shown in Table 1. Participants diagnosed with liver cancer were older and were more likely to be male; be a current or passive smoker; consume alcohol; have a higher BMI; be positive for the hepatitis B surface antigen (HBsAg); or have a medical history of hepatobiliary disease, hypertension, or diabetes (all *P* values <.05; Table 1).

The associations between various factors and the risk of liver cancer are shown in Table 1. Age, sex, BMI, smoking, alcohol consumption, HBsAg status, chronic HBV infection, chronic HCV infection, cirrhosis, a history of schistosomiasis infection, hypertension, and diabetes were positively associated with liver cancer, while education level was inversely associated with liver cancer. The associations of liver cancer with occupational exposure to hazardous substances, frequent exercise, family history of liver cancer, fatty liver disease, gallstones, and hyperlipidemia were not statistically significant.

**Table 1.** Baseline characteristics and their associations with liver cancer.

Baseline characteristics	Total (n=153,082)	Non–liver cancer (n=152,792)	Liver cancer (n=290)	*P* value	HR^a^ (95% CI)^b^	*P* value
Demographic characteristics						
Age (years), mean, (SD)	55.86 (8.43)	55.85 (8.43)	59.20 (7.15)	<.001	1.06 (1.05‐1.08)	<.001
Sex, n (%)				<.001		
Female	85,496 (55.8)	85,416 (55.9)	80 (27.6)		1.0 (reference)	
Male	67,586 (44.2)	67,376 (44.1)	210 (72.4)		3.38 (2.62‐4.38)	<.001
Education level, n (%)				.12		
Low	50,410 (32.9)	50,303 (32.9)	107 (36.9)		1.0 (reference)	
Medium	86,145 (56.3)	85,984 (56.3)	161 (55.5)		0.77 (0.60‐0.98)	.04
High	16,527 (10.8)	16,505 (10.8)	22 (7.6)		0.52 (0.33‐0.83)	.006
BMI, n (%)				<.001		
<25	70,099 (45.8)	69,997 (45.8)	102 (35.2)		1.0 (reference)	
≥25	28,056 (18.3)	27,994 (18.3)	62 (21.4)		1.49 (1.09‐2.05)	.01
Missing	54,927 (35.9)	54,801 (35.9)	126 (43.4)		1.12 (0.86‐1.46)	.39
Lifestyle factors						
Occupational exposure to hazardous substances, n (%)				.37		
No	132,641 (86.6)	132,384 (86.6)	257 (88.6)		1.0 (reference)	
Yes	20,441 (13.4)	20,408 (13.4)	33 (11.4)		0.91 (0.63‐1.31)	.61
Smoking, n (%)				<.001		
Never	112,581 (73.5)	112,427 (73.6)	154 (53.1)		1.0 (reference)	
Current	31,873 (20.8)	31,765 (20.8)	108 (37.2)		2.45 (1.92‐3.13)	<.001
Former	8628 (5.6)	8600 (5.6)	28 (9.7)		2.42 (1.62‐3.62)	<.001
Alcohol consumption, n (%)				<.001		
Never	109,243 (71.4)	109,077 (71.4)	166 (57.2)		1.0 (reference)	
Current	38,465 (25.1)	38,367 (25.1)	98 (33.8)		1.67 (1.30‐2.14)	<.001
Former	5372 (3.5)	5346 (3.5)	26 (9)		3.17 (2.10‐4.79)	<.001
Missing	2 (0)	2 (0)	0 (0)		—	—
Frequent exercise, n (%)				.48		
No	82,590 (54)	82,440 (54)	150 (51.7)		1.0 (reference)	
Yes	70,492 (46)	70,352 (46)	140 (48.3)		1.00 (0.79‐1.26)	>.99
Family history of liver cancer, n (%)				.19		
No	142,572 (93.1)	142,308 (93.1)	264 (91)		1.0 (reference)	
Yes	10,510 (6.9)	10,484 (6.9)	26 (9)		1.45 (0.97‐2.18)	.07
Hepatobiliary disease						
HBsAg^c^, n (%)				<.001		
Negative	36,493 (23.8)	36,425 (23.8)	68 (23.4)		1.0 (reference)	
Positive	5917 (3.9)	5860 (3.8)	57 (19.7)		5.70 (4.01‐8.11)	<.001
Unknown	110,672 (72.3)	110,507 (72.3)	165 (56.9)		0.99 (0.74‐1.31)	.92
Chronic hepatitis B infection, n (%)				<.001		
No	148,446 (97)	148,195 (97)	251 (86.6)		1.0 (reference)	
Yes	4636 (3)	4597 (3)	39 (13.4)		5.01 (3.57‐7.02)	<.001
Chronic hepatitis C infection, n (%)				.04		
No	152,424 (99.6)	152,138 (99.6)	286 (98.6)		1.0 (reference)	
Yes	658 (0.4)	654 (0.4)	4 (1.4)		3.59 (1.34‐9.64)	.01
Cirrhosis, n (%)				<.001		
No	152,028 (99.3)	151,770 (99.3)	258 (89)		1.0 (reference)	
Yes	1054 (0.7)	1022 (0.7)	32 (11)		20.20 (14.00‐29.20)	<.001
History of schistosomiasis infection, n (%)				.06		
No	149,581 (97.7)	149,303 (97.7)	278 (95.9)		1.0 (reference)	
Yes	3501 (2.3)	3489 (2.3)	12 (4.1)		2.08 (1.17‐3.71)	.01
Fatty liver, n (%)				.33		
No	127,924 (83.6)	127,675 (83.6)	249 (85.9)		1.0 (reference)	
Yes	25,158 (16.4)	25,117 (16.4)	41 (14.1)		0.86 (0.62‐1.19)	.37
Gallstones, n (%)				.54		
No	140,503 (91.8)	140,242 (91.8)	261 (90)		1.0 (reference)	
Yes	12,574 (8.2)	12,545 (8.2)	29 (10)		1.26 (0.86‐1.85)	.24
Missing	5 (0)	5 (0)	0 (0)		—	—
Systemic diseases						
Hypertension, n (%)				.01		
No	111,191 (72.6)	111,003 (72.6)	188 (64.8)		1.0 (reference)	
Yes	41,890 (27.4)	41,788 (27.3)	102 (35.2)		1.46 (1.15‐1.86)	.002
Missing	1 (0)	1 (0)	0 (0)		—	—
Hyperlipidemia, n (%)				.15		
No	128,594 (84)	128,341 (84)	253 (87.2)		1.0 (reference)	
Yes	24,488 (16)	24,451 (16)	37 (12.8)		0.76 (0.54‐1.07)	.11
Diabetes, n (%)				<.001		
No	140,855 (92)	140,609 (92)	246 (84.8)		1.0 (reference)	
Yes	12,227 (8)	12,183 (8)	44 (15.2)		2.08 (1.51‐2.87)	<.001

a HR: hazard ratio.

b Hazard ratios were not calculated for missing values.

c HBsAg: hepatitis B surface antigen.

### Prediction Model Development and Evaluation

**Figure 1.**
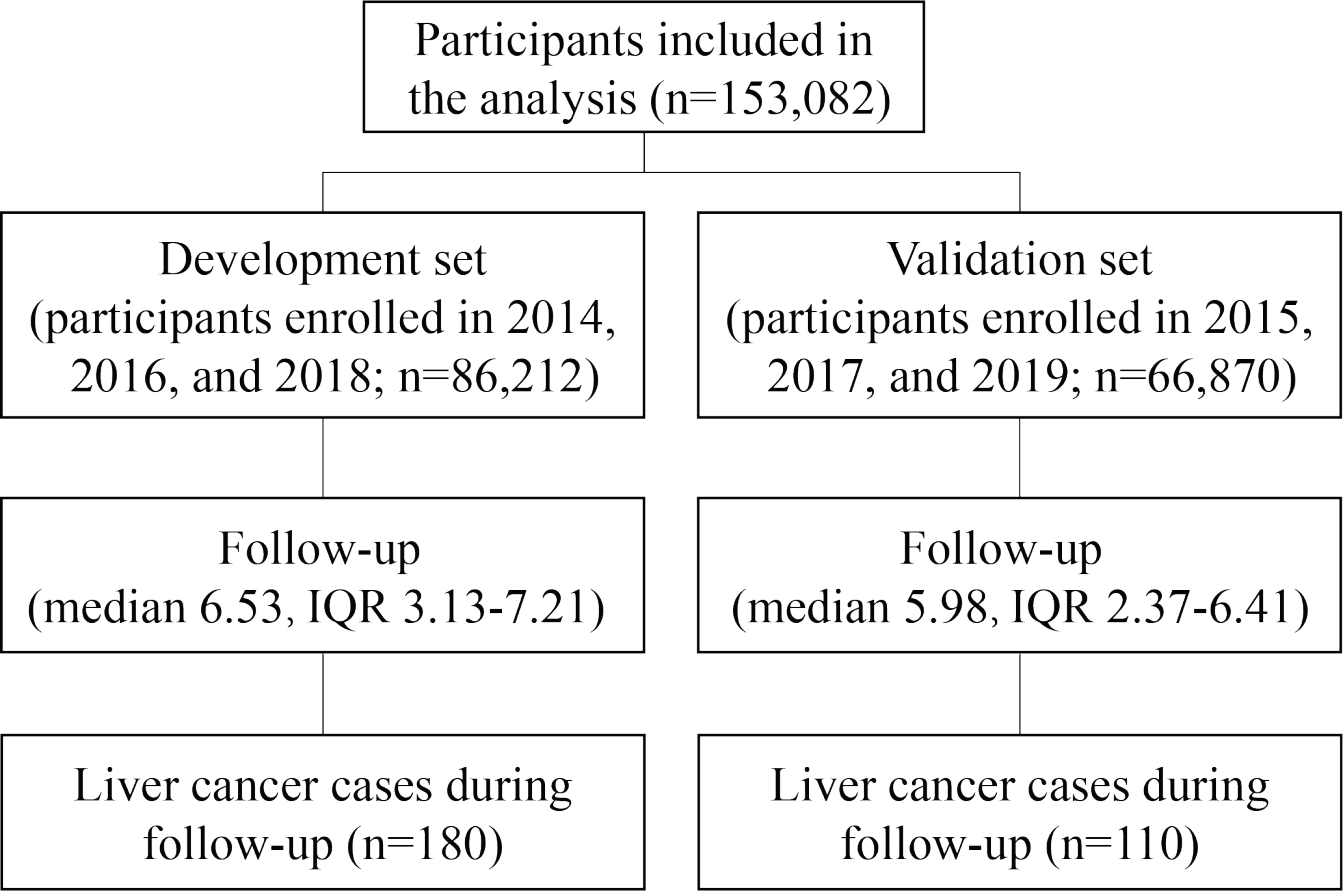
Development and validation sets of the prediction model.

**Figure 2.**
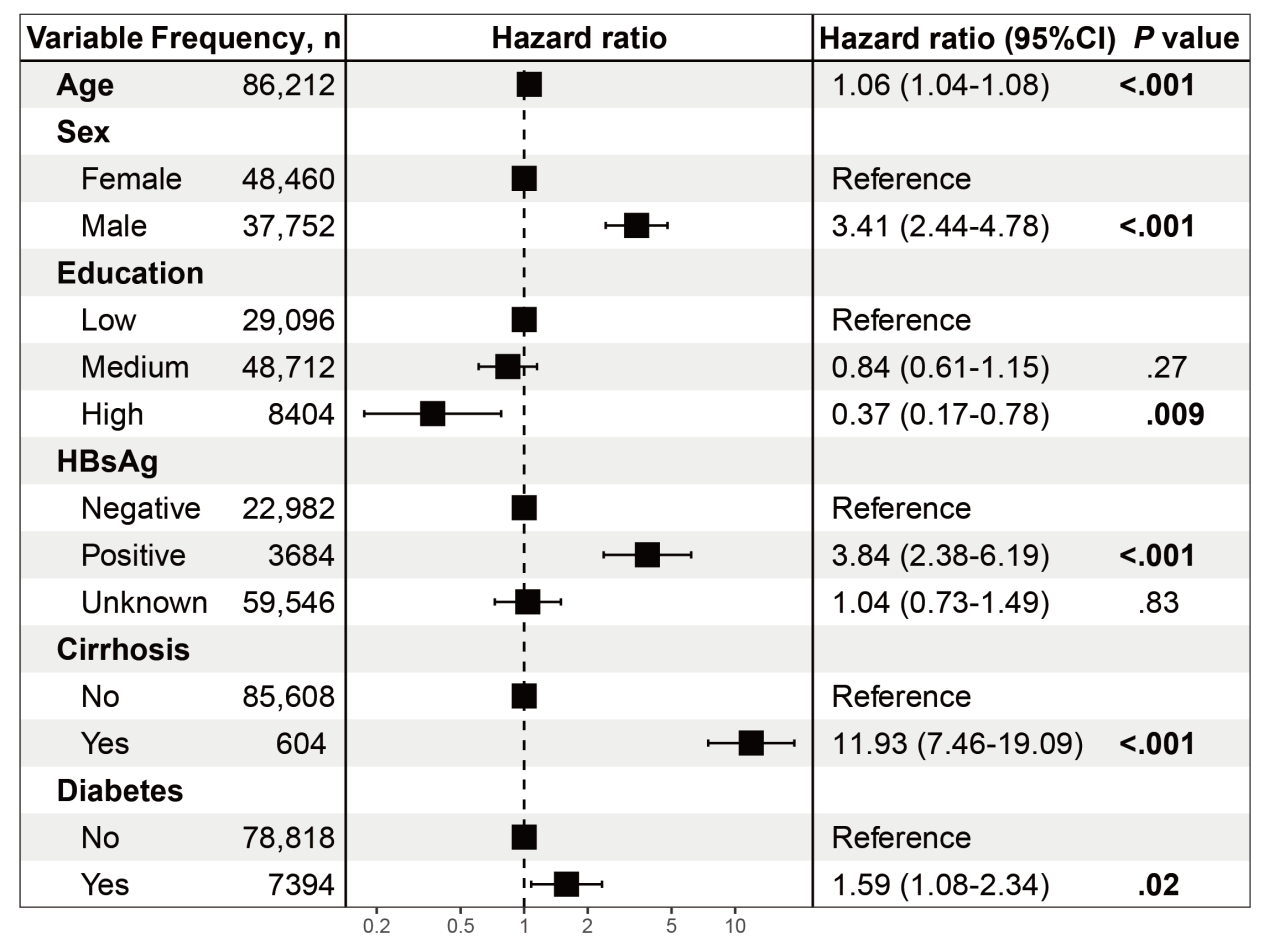
Adjusted hazard ratios and 95% CIs of liver cancer risk factors from a multivariable Cox regression model in the development set. HBsAg: hepatitis B surface antigen.

**Figure 3.**
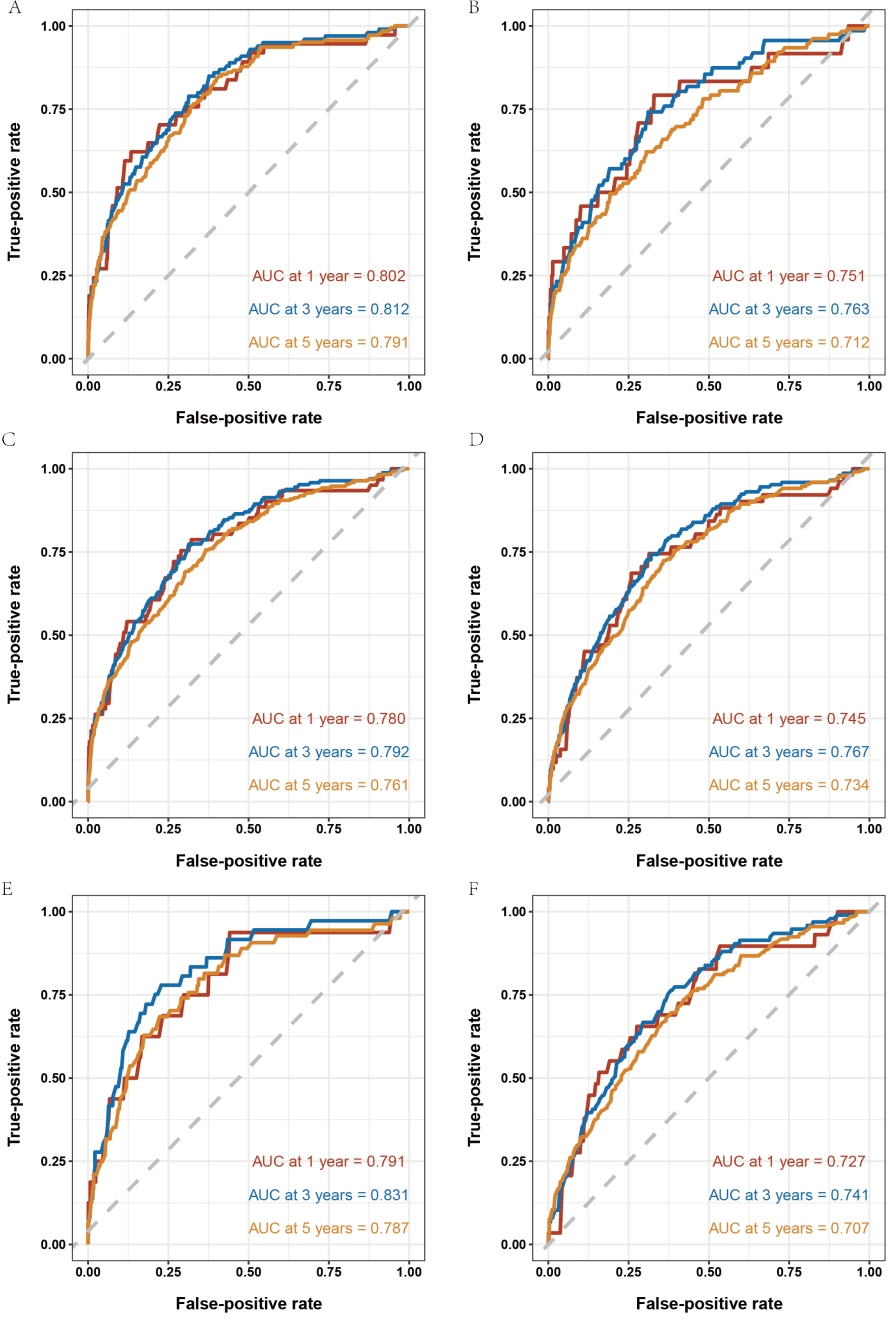
The receiver operating characteristic curves of the prediction model in different groups. (**A**) Development set. (**B**) Validation set. (**C**) Whole population. (**D**) Participants without cirrhosis. (**E**) Hepatitis B surface antigen negative group and (**F**) Hepatitis B surface antigen unknown group. AUC: area under the curve.

We divided the entire dataset by year of enrollment to develop and internally validated the prediction model (Figure 1). The model was developed using data from the CanSPUC, with 86,212 participants enrolled in 2014, 2016, and 2018. During a median follow-up of 6.53 (IQR 3.13‐7.21) years, 180 participants were diagnosed with liver cancer. The 6 potential covariates of age, sex, education level, cirrhosis, diabetes, and HBsAg status were included in the final Cox regression model after backward selection. The hazard ratios and 95% CIs for the risk model based on development dataset are listed in Figure 2. A multivariable-adjusted analysis showed that age, male sex, HBsAg positivity, the presence of cirrhosis, and diabetes were independently associated with an increased risk of liver cancer, while a higher education level was independently associated with a reduced risk. In the development set, the model’s performance was indicated by the AUC of the 1-, 3-, and 5-year risk of liver cancer, which were 0.802, 0.812, and 0.791, respectively (Figure 3A). We also plotted 1-, 3-, and 5-year liver cancer risk prediction nomogram (Multimedia Appendix 1). The validation set included participants from the CanSPUC enrolled in 2015, 2017 and 2019. These participants were followed for a median of 5.98 (IQR 2.37‐6.41) years, and 110 cases of liver cancer occurred. When applied to the validation set and the entire population, the model was able to discriminate well between participants with and without liver cancer in 1-, 3- and 5-year intervals, with the AUC ranging from 0.712 to 0.792 (Figure 3B-C). Sensitivity analyses applying the model to participants without cirrhosis and a HBsAg-negative or unknown status yielded similar performances, with the AUC ranging from 0.707 to 0.831 (Figure 3D-F). The calibration plots indicated an excellent agreement between the observed and predicted probabilities of developing liver cancer over 1-, 3-, and 5-year periods (MultimediaAppendices 2  3).

### Risk Score for Liver Cancer

**Figure 4.**
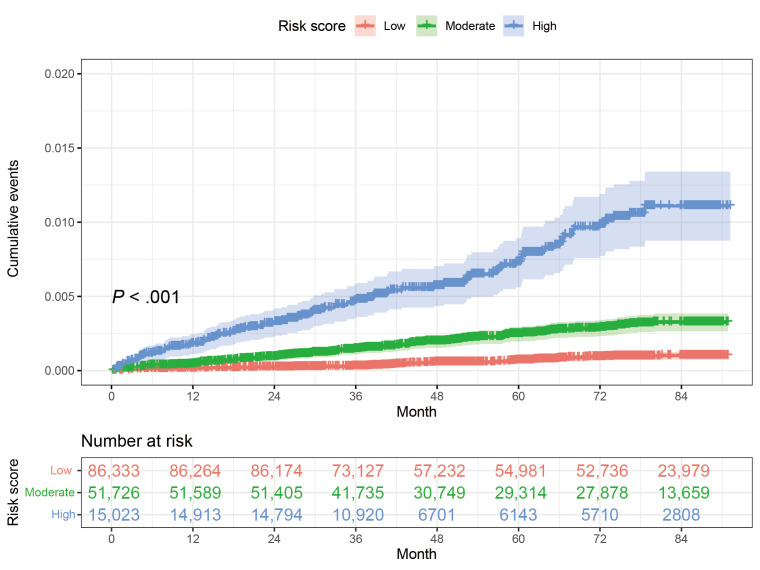
Cumulative incidence of liver cancer in the whole population.

**Figure 5.**
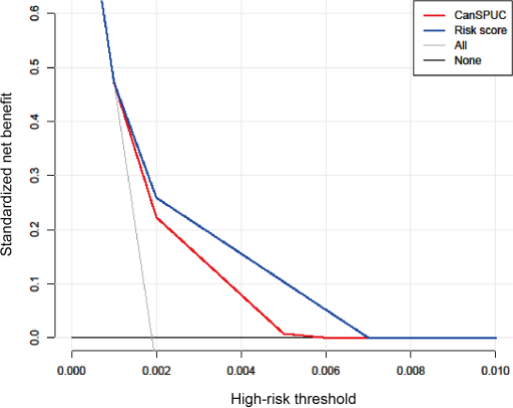
Decision curves of risk score compared to the CanSPUC strategy. CanSPUC, Cancer Screening Program in Urban China.

The risk score was derived using 6 variables, weighted by the β coefficients from the multivariable model and then standardized (Table 2). Participants were subsequently divided into 3 groups according to the risk score: low-, moderate-, and high-risk for liver cancer. The cutoff values of the risk score (30.2 and 44.3) were chosen by the X-tile plots. A significant gradient in liver cancer was observed across the risk score categories. Compared to those with a low-risk score, the relative risk of liver cancer was 3.51-fold and 11.88-fold higher for participants with moderate-risk or high-risk scores (moderate-risk: HR 3.51, 95% CI 2.58‐4.76; high-risk: HR 11.88, 95% CI 8.67‐16.27). The cumulative liver cancer incidences stratified by risk score (*P*<.001) are shown in Figure 4. The decision curve analysis showed that the risk score provided a higher net benefit compared to the current CanSPUC strategy (Figure 5). The risk score provided a practical tool for liver cancer surveillance among the general population. To aid in individual risk stratification, a user-friendly web-based scoring system was developed using routine parameters (age, sex, education level, cirrhosis, diabetes, and HBsAg status) [24].

**Table 2.** Risk sore assignment.

Variable	Score
Age	(Age − 40) × 0.056
Sex	
Female	0
Male	1.23
Education	
Low	0
Medium	−0.18
High	−0.998
HBsAg^a^ status	
Negative	0
Positive	1.35
Unknown	0.04
Cirrhosis	
No	0
Yes	2.48
Diabetes	
No	0
Yes	0.46

a HBsAg: hepatitis B surface antigen.

## Discussion

### Principle Findings

In this large population-based prospective cohort study, we identified factors associated with the risk of liver cancer and developed a prediction model along with a risk scoring system to assist in self-assessments and the stratification of the population for liver cancer screening. When assessing participants’ risk of liver cancer at 1-, 3- and 5-year periods, the model demonstrated a high level of calibration and discriminative ability. The model was validated internally and across subgroups without cirrhosis and with an HBsAg-negative or unknown status, showing similar performance. Compared to the low-risk score group, participants in the moderate-risk and high-risk score groups exhibited 3.51-fold and 11.8-fold higher risks of liver cancer, respectively.

We comprehensively analyzed the associations between epidemiological characteristics, lifestyle factors, and general health indicators with liver cancer risk through a large longitudinal follow-up cohort study. Among the factors associated with liver cancer, age, sex, education level, cirrhosis, diabetes, and HBsAg status were found to be critical for evaluating liver cancer risk. The epidemiology of liver cancer is characterized by significant demographic variations [25]. Age is a well-known risk factor for liver cancer. According to data from the National Cancer Center of China [26], the incidence of liver cancer increases rapidly after the age of 30, particularly in men. This may be due to the accumulation of damage to liver cells, changes in the immune system, and the development of chronic liver disease over time. Our results suggest that higher education level reduces the risk of liver cancer, likely because of the increased awareness and knowledge about health-related behaviors and risk factors. However, the association between education level and liver cancer risk remains unclear, as findings vary by sex, region, and study design [27,28]. Other factors, such as income, occupation, and access to health care, may also influence the relationship between education and liver cancer risk, which could contribute to the inconsistent results in the literature.

Cirrhosis, a progressive and irreversible disease in which liver cells are damaged and replaced by scar tissue, is a key risk factor for liver cancer. In fact, most cases of liver cancer occur in individuals with underlying cirrhosis. Regular screening for liver cancer is recommended for individuals with cirrhosis, as early detection can improve treatment outcomes. Recently, an increasing trend in liver cancer rates has been reported in several developed countries in Europe and North America [25]. These new trends are associated with recently determined risk factors, such as HCV infection [29] and possibly diabetes [30,31]. Our study showed that individuals with diabetes had a 2.08-fold higher risk of liver cancer. This increased risk may be attributed to several factors, including insulin resistance, inflammation, and oxidative stress, which are common features in both diabetes and liver cancer [32].

Additionally, our study found that being overweight and consuming alcohol were both associated with an increased risk of liver cancer. Several mechanisms have been proposed to explain the association between being overweight or obese and liver cancer, including insulin resistance, chronic inflammation, and the accumulation of fat in the liver. Being overweight or obese can also lead to nonalcoholic fatty liver disease, which can progress to a more severe form called nonalcoholic steatohepatitis [33], ultimately increasing the risk of liver cancer. Chronic alcohol consumption is another key risk factor, as it can lead to liver cirrhosis, a major risk factor of liver cancer. A meta-analysis has shown that heavy alcohol consumption is associated with an increased risk of liver cancer compared to nondrinkers, with the association being dose-dependent [34]. Smoking was also identified as risk factor of liver cancer, consistent with previously reported studies [35,36]. Individuals who have quit smoking exhibit a reduced risk of liver cancer compared to those who continue to smoke. However, both groups still have a higher risk of liver cancer compared to individuals who have never smoked. In summary, these risk factors collectively increase the risk of liver cancer. Therefore, preventive measures such as hepatitis B vaccination, limiting alcohol consumption, maintaining a healthy weight, and smoking cessation can help reduce the risk of liver cancer.

Available liver cancer risk prediction models have primarily focused on high-risk populations, such as the THRI (Toronto HCC Risk Index) [5] and GES (General Evaluation Score) [6] for patients with cirrhosis and the REACH-B (Risk Estimation for Hepatocellular Carcinoma in Chronic Hepatitis B) [7], PAGE-B (Platelets, Age, Gender, and HBV) [8], AGED (Age, Gender, HBV e antigen, and HBV DNA) [9], and aMAP (Age, Male, Albumin-bilirubin, and Platelets) [14] for individuals with HBV infection, HCV infection, and chronic hepatitis with different etiologies. Currently, screening strategies for the general population are not recommended by guidelines in most countries. The cancer risk assessment questionnaire used in the CanSPUC program is a joint screening tool for the 5 common cancers, with the reference conditions for defining high-risk liver cancer groups being men aged 45‐74 years; women aged 50‐74 years; individuals who are HBsAg-positive; those with a history of HCV infection or cirrhosis; or those with a family history of liver cancer in first- or second-degree relatives. By collecting multiple variables from this large cohort study, we developed an accurate and personalized prediction model and risk score for assessing liver cancer risk. The China Kadoorie Biobank collaborative group developed a prediction model [37] based on 500,000 community residents, integrating 15 items across 6 dimensions, including demographic characteristics, behavioral and lifestyle factors, personal medical history, family medical history, body measurements, and blood test results. Our model was able to simplify the prediction process by focusing on 6 common variables, which are routinely available. Specifically, HBsAg status can be easily obtained through regular physical checkups or blood tests. Our study provides a valuable model for identifying high-risk populations for liver cancer, which can be effectively used in screening and surveillance efforts.

### Limitations

There are several limitations to this study. First, the study population may not fully represent the general population of China, but it can serve as a reference for similar socioeconomic regions. Second, this study focused on individuals aged 40‐74 years. Thus, the risk factors for younger populations require further investigation. Future studies should aim to include a broader age range to enhance the applicability of the model to other populations, such as cohort studies based on regular health examination programs. Third, with a median follow-up of 6.07 years, the duration may not be sufficient to capture all liver cancer cases. Extended follow-up would be needed to confirm the long-term effectiveness and reliability of the model. Fourth, due to the limited data availability, the risk factors for specific subtypes of liver cancer should be investigated in the future studies. Fifth, although we performed extensive internal validation and subgroup analysis, external validation in independent populations is necessary to ensure the model’s robustness and refine prevention strategies for larger populations. Furthermore, the model’s predictive accuracy could be improved with mechanistic experimental research that identifies specific biological pathways and molecular markers for liver cancer development. This would help to deepen our understanding of liver cancer pathogenesis and optimize risk predictions based on mechanistic insights.

### Conclusions

In conclusion, we developed a liver cancer risk prediction model that incorporates age, sex, education level, cirrhosis, diabetes, and HBsAg status. This model offers a user-friendly tool for residents to assess their risk of developing liver cancer using commonly available parameters. The model has the potential to optimize liver cancer screening programs, significantly improving the effectiveness of early detection and self-assessment, thus contributing to better liver cancer prevention and surveillance efforts.

## Supplementary material

10.2196/65286 Multimedia Appendix 1 Nomogram to calculate the personal 1-, 3-, and 5-year risk of liver cancer.

10.2196/65286 Multimedia Appendix 2 Calibration curves of the prediction model for (A) 1-year, (B) 3-year, and (C) 5-year liver cancer risk in the development set.

10.2196/65286 Multimedia Appendix 3 Calibration curves of the prediction model for (A) 1-year, (B) 3-year, and (C) 5-year liver cancer risk in the validation set.

## Abbreviations

AGED: Age, Gender, HBV e antigen, and HBV DNA
aMAP: Age, Male, Albumin-bilirubin, and Platelets
AUC: area under the curve
CanSPUC: Cancer Screening Program in Urban China
GES: General Evaluation Score
HBsAg: hepatitis B surface antigen
HBV: *Hepatitis B virus*
HCV: *Hepatitis C virus*
HR: hazard ratio
PAGE-B: Platelets, Age, Gender, and HBV
REACH-B: Risk Stimation for Hepatocellular Carcinoma in Chronic Hepatitis B
ROC: receiver operating characteristic
THRI: Toronto HCC Risk Index
