# Supplementary figures and images for: Development of a Prediction Model and Risk Score for Self-Assessment and High-Risk Population Identification in Liver Cancer Screening: Prospective Cohort Study
Source: JMIR Public Health Surveill. 2024 Dec 30;10:e65286. doi: 10.2196/65286 (PMC11702484; doi:10.2196/65286)

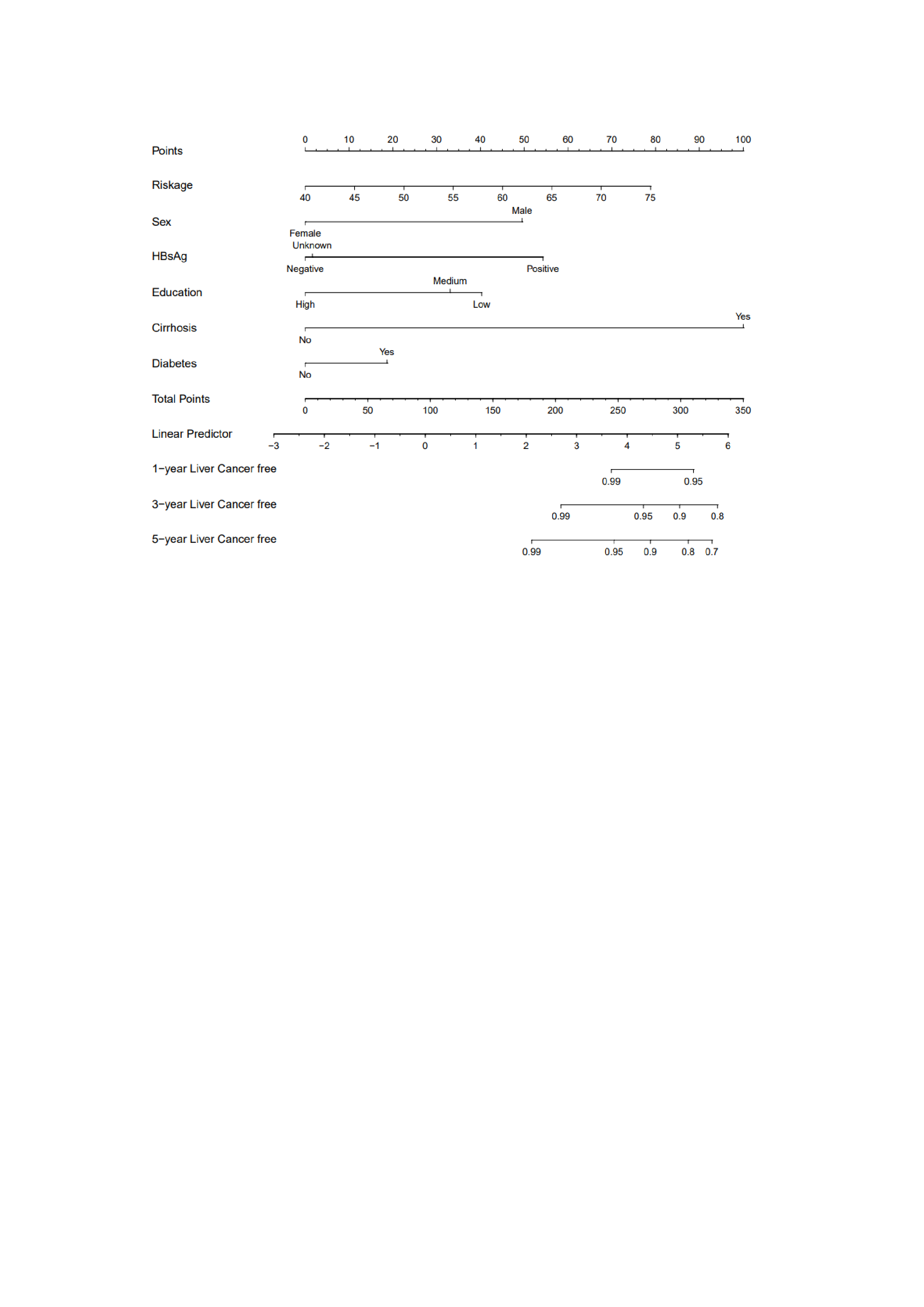

Supplement: Multimedia Appendix 1 [file publichealth-v10-e65286-s001.png]

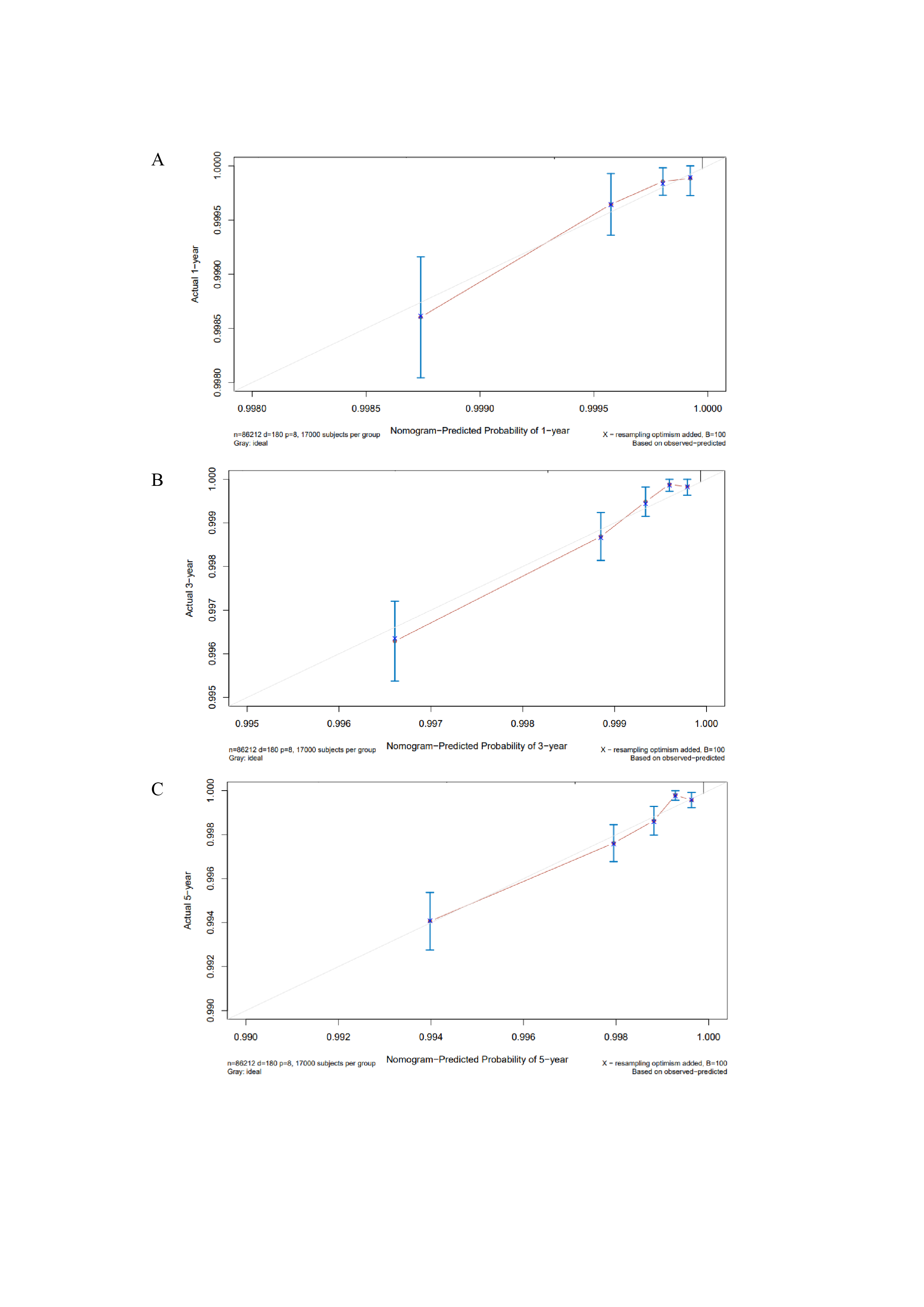

Supplement: Multimedia Appendix 2 [file publichealth-v10-e65286-s002.png]

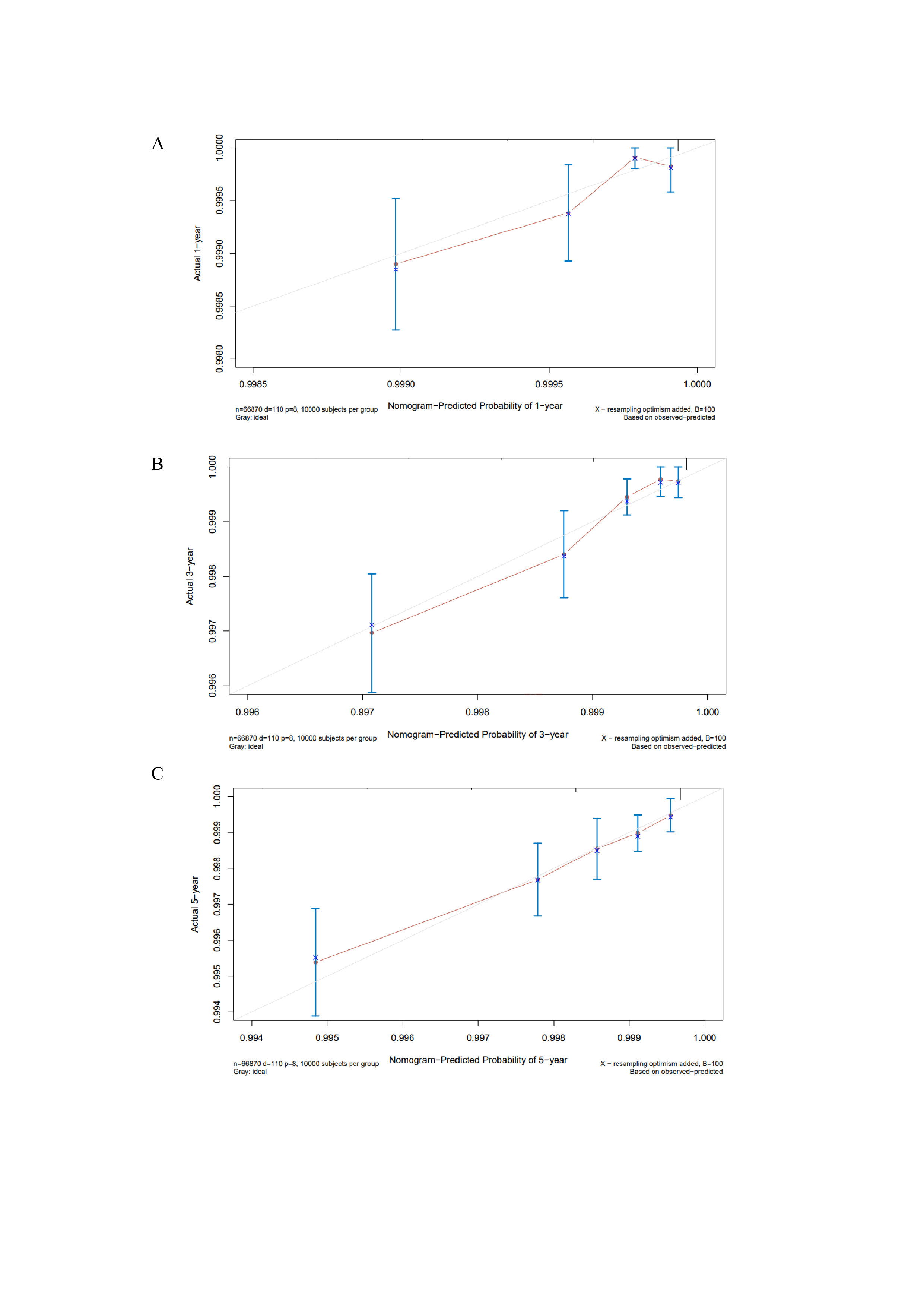

Supplement: Multimedia Appendix 3 [file publichealth-v10-e65286-s003.png]
